# Chronic kidney disease (CKD) patients are exposed to more proton pump inhibitor (PPI)s compared to non-CKD patients

**DOI:** 10.1371/journal.pone.0203878

**Published:** 2018-09-13

**Authors:** Hee Jeong Lee, Haekyung Lee, Song Hee Oh, Joonbyung Park, Suyeon Park, Jin Seok Jeon, HyunJin Noh, Dong Cheol Han, Soon Hyo Kwon

**Affiliations:** 1 Department of Internal medicine, Soonchunhyang University Seoul Hospital, Seoul, Korea; 2 Department of Biostatistics, Soonchunhyang University Seoul Hospital, Seoul, Korea; 3 Hyonam Kidney Laboratory, Soonchunhyang University Seoul Hospital, Seoul, Korea; Hospital Universitario de la Princesa, SPAIN

## Abstract

**Background:**

Proton pump inhibitor use is associated with incident chronic kidney disease, chronic kidney disease progression and end-stage renal disease. However, the extent of proton pump inhibitor prescriptions to chronic kidney disease patients is still unclear.

**Method:**

In a retrospective study, we enrolled patients (>18 years old) who received proton pump inhibitor in the out-patient setting from 2014 through 2015. All data was obtained from electronical medical records of Soonchunhyang Medical Center. The prescription patterns and characteristics of proton pump inhibitors were analyzed according to individual estimated glomerular filtration rate of the patients.

**Result:**

During the study period 178,228 patients visited the out-patient clinic. Proton pump inhibitors were prescribed to 9,109 (5.11%) patients. In our sample, 50% were females and 9.8% were chronic kidney disease (eGFR<60mL/min/1.73 m^2^) patients. Among the patients with chronic kidney disease, 730 (8.0%) were categorized as stage 3 or 4 and 166 (1.8%) were categorized as stage 5 or end-stage renal disease. The prevalence of proton pump inhibitors prescription among chronic kidney disease patients was higher than in the non-chronic kidney disease group (p<0.001). Median duration of usage was 120 [interquartile range 63–273] days in the stage 3–4 group, 106 [56–266] days in the stage 5-end-stage renal disease group and 90 [56–176] days in the non-chronic kidney disease group. Patients in stage 3–4 group were prescribed longer duration of proton pump inhibitors than the non-chronic kidney disease group even after adjusting for age and sex (p<0.001). The main departments of medicine which prescribed proton pump inhibitors for the stage 3–4 group were gastroenterology (40.0%), cardiology (29.6%), nephrology (9.5%) and neurology (4.8%). Compared to the non-chronic kidney disease group, the stage 3–4 and stage 5-end-stage renal disease group were taking larger number of drugs simultaneously (6.90±4.17 vs4.54±2.43; p<0.001, 5.64±2.87 vs 4.54±2.34; p<0.001, respectively).

**Conclusion:**

Chronic kidney disease patients are taking proton pump inhibitors for a much longer duration compared to non-chronic kidney disease patients. Physicians should pay careful attention when prescribing proton pump inhibitors to high risk groups.

## Introduction

Proton pump inhibitor (PPI)s are one of the most commonly prescribed medications for the treatment of reflux esophagitis, dyspepsia and peptic ulcer disease [1]. PPIs block the H+/K+ ATPase enzyme in gastric wall cells, leading to inhibition of acid secretion. However, they are often overprescribed and approximately 25% to 70% of these prescriptions have no appropriate indication [2].

Generally, PPIs are perceived as safe, but several observational studies have linked PPI use to uncommon but serious adverse health outcomes, such as hip fracture, community acquired pneumonia, clostridium difficile infections and incident dementia [3–5].

PPIs undergo hepatic metabolism and do not require dosage modification in patients with kidney disease. However, PPIs are recognized as one of the most common causes of drug-induced acute interstitial nephritis (AIN) and acute kidney injury [6–9]. Furthermore, treatment with PPI was associated with significantly elevated risk of doubling of serum creatinine (Cr) level, of decline over 30% of estimated glomerular filtration rate (eGFR) [10] and of progression to end-stage renal disease (ESRD) independent of AIN [11]. In addition, there was graded association between longer duration of PPI exposure as well as higher dosage of PPI and higher risk of chronic kidney disease (CKD) [10]. Furthermore, a longitudinal observational cohort study showed PPI use was also associated with increased risk of death compared with H2 blocker use [12].

Therefore, prescription patterns may have clinical significance in preventing excessive prescription of PPIs in the high risk group. CKD patients may have various indications of CKD. Gastric ulcers and gastroesophageal reflux disease (GERD) are more prevalent in those with CKD compared to those without. However, there has been no previous study examining the prescription patterns of PPIs in CKD. Greater exposure to PPIs can be a risk for CKD patients. The objective of this study was to compare the extent of PPI prescription according to CKD stage to determine the amount of exposure patients with CKD had.

## Materials and methods

### Study design

We retrospectively reviewed medical records of patients (>18 years old) who received any PPIs in the outpatient setting from January 2014 to December 2015. The data was obtained from electronic medical records from Soonchunhyang Medical Clinic. The study protocol was approved by the institutional review board (IRB) of Soonchunhyang Hospital (IRB number 2017-05-001) and conducted in accordance with the Declaration of Helsinki. This was an anonymous observational study, so the need for informed consent was waived. Patient diagnoses were based on International Statistical Classification of Diseases and Related Health Problems 10th Revision (ICD-10-CM) diagnostic and procedure codes. Subjects were limited to outpatients because inpatients are often referred from the local hospitals, so continuous follow up of treatment was not able.

### Data collection

Patient’s clinical data was extracted based on the date the patients were given the longest duration of PPIs. Because a doctor’s prescription is needed for all PPIs in Korea under the drug utilization review (DUR) system, repeated prescription of PPIs from different doctors are very rare. Data on age, sex, chronic illnesses (including hypertension, diabetes mellitus, cardiovascular disease, chronic obstructive pulmonary disease, congestive heart failure, liver cirrhosis), the department the patient visited, the generic name of the PPI, and the main diagnosis at visit were obtained. All disease and health states were based on ICD-10 codes in the electronic medical record.

For laboratory tests, blood urea nitrogen (BUN), Cr and estimated glomerular filtration rate (eGFR calculated from CKD-EPI equation) [13] data was obtained. The patients were divided into three groups, according to their eGFR. The first group was the non CKD group, consisting of CKD stage 1–2 patients (eGFR≥60mL/min/1.73m^2^) by the Kidney disease: Improving global outcomes (KDIGO) 2012 guidelines. The second group was the CKD stage 3–4 group, which included patients with 15≤ eGFR <60mL/min/1.73m^2^. And the third group was the CKD stage 5-ESRD group and included patients eGFR<15mL/min/1.73m^2^ or those on dialysis.

#### Exposure to PPIs and prescription patterns

To assess exposure to PPIs, we looked at total duration of PPI administration during the observation period. Total duration of PPIs was calculated by summing up the duration of PPI prescriptions from each outpatient visit during the study period. We also investigated the longest duration of PPI prescription given at one outpatient clinic visit. The medicines prescribed simultaneously with PPIs were classified into three groups; non-steroidal anti-inflammatory drugs (NSAIDs), steroids and anticoagulant/antiplatelet agents. The number of patients in each classification was counted. We also recorded the department from which patients received their PPI prescriptions and analyzed differences between the three groups.

### Statistical analyses

Baseline characteristic comparisons across multiple groups were performed using Mann Whitney test or Kruskal-Wallis test for continuous variables and chi-square tests or the Fisher exact test for categorical variables. Post-hoc test was performed and bonferroni correction method was used to solve the multiple testing problems. To identify the association between the CKD groups and the duration of PPI administration, we used two-stage analysis. First, we calculated the weight variable for adjusting for age and sex for each group [14]. Second, a generalized linear model with inverse-probability weighting was used to demonstrate the relationship between CKD groups and duration of PPI use, and the p-value was estimated by the likelihood ratio test [15, 16]. In the generalized linear model, we assumed a Gaussian distribution, and we used the “identity” as the link function. Pair-wised comparisons were performed and p-value adjusted by bonferroni correction. In correction analysis, patients without eGFR data in non CKD group were excluded. Data are presented as means ± standard deviation, or medians with interquartile range [IQR] if variables showed non-normal distribution. Nominal data are shown as percentages. All statistical analyses were performed with the SPSS version 22.0 (SPSS Inc, Chicago, IL, USA) and R program. A two-sided p<0.05 was considered to indicate statistical significance in most cases. However, p<0.001 was considered as significance in bonferroni correction.

## Results

### Characteristics

During study period, 178,228 patients visited our hospital out-patient clinic. Of the total patients 5,874 (3.29%) were CKD patients. PPIs were prescribed to 9,109 patients (5.11%) and 730 CKD patients were prescribed PPIs, which accounted for 12.42% of CKD patients. In the out-patient clinic, the prevalence of PPI prescription in CKD patients was higher than the non CKD group (p<0.001). Among patients who took PPIs, 50.3% were female and 9.8% were CKD (eGFR<60mL/min/1.73 m^2^) patients. Among CKD patients, 730 (8.0%) were categorized as stage 3 or 4 and 166 (1.8%) were stage 5 or ESRD. Patients in the CKD stage 3–4 group and CKD stage 5-ESRD group had higher percentage of hypertension, diabetes and congestive heart failure. The demographic and health characteristics of the groups are described in Table 1.

**Table 1:** Baseline characteristics of CKD stage 3–4, CKD stage 5-ESRD, and non-CKD groups.

Characteristi*c*s	Non-CKD group (A)	CKD stage 3–4 group (B)	CKD stage 5-ESRD (C)	P value	Post-hoc†
Total	8213 (90.2%)	730 (8.0%)	166 (1.8%)		
Mean age (years)	58.08±14.15	72.30±12.10	62.45±13.20	0.0001	A<C<B
Gender (Male %)	4115 (50.1%)	331 (45.3%)	80 (48.1%)	0.044	A>B>C
Presence of chronic illness (% by groups)					
THTN	1864 (22.7%)	413 (56.6%)	95 (57.2%)	<0.0001	A<B<C
DDM	1462 (17.8%)	300 (41.1%)	83 (50.0%)	<0.0001	A<B<C
CCVD	987 (12.0%)	216 (29.6%)	51 (30.7%)	<0.0001	A<B<C
LLC	179 (2.2%)	28 (3.8%)	9 (5.4%)	0.001	A<B<C
CCHF	109 (1.3%)	70 (9.6%)	17(10.2%)	0.0001	A<B<C
CCOPD	78 (0.9%)	17 (2.3%)	2 (1.2%)	0.002	A<C<B
eGFR (ml/min/1.73 m^2^)	92.78±14.86	44.49±12.06	7.33±3.19	<0.0001	C<B<A

CKD, chronic kidney disease; ESRD, end-stage renal disease; eGFR, estimated glomerular filtration rate; HTN, hypertension; DM, diabetes mellitus; CVD, cardiovascular disease; LC, liver cirrhosis; CHF, congestive heart failure; COPD, chronic obstructive pulmonary disease. eGFR was calculated using CKD-EPI formula. Age and eGFR are presented as mean ± standard deviation (SD). Nominal data are presented as percentages

†p-value by student t-test or chi-squared/fisher exact test and adjusted by bonferroni correction

### PPIs prescribed and indications for prescription

Eight different PPIs were prescribed. The most commonly used PPI was lansoprazole, which was prescribed in 2,626 (28.8%) cases followed by pantoprazole in 1,556 (17.1%) (Table 2). Only one PPI was a complex agent combined with NSAIDs. The most common diagnostic code related to indications for PPI was reflux esophagitis, which was found in 8.2% of the patients. Early gastric cancer with mucosal dissection (2.8%) was the next most frequent, and eradication of Helicobacter pylori infection the third (1.8%). Based on the Food and Drug Administration’s (FDA) approved indications list [17], 2,492 (27.3%) patients had appropriate indications for a PPI.

**Table 2:** Proton-pump inhibitors used in the out-patient clinic.

Type	Non-CKD group (A)	CKD stage 3–4 group (B)	CKD stage 5-ESRD (C)
Lansoprazole	2294 (27.9%)	271 (37.1%)	61 (36.8%)
Pantoprazole	1381 (16.8%)	147 (20.1%)	28 (16.9%)
Esomeprazole	1366 (16.6%)	127 (17.4%)	40 (24.1%)
Ilaprazole	1406 (17.1%)	68 (9.3%)	15 (9.0%)
Rabeprazole	1262 (15.4%)	65 (8.9%)	9 (5.4%)
Dexlansoprazole	424 (5.2%)	48 (6.6%)	4 (2.4%)
Revaprazan	63 (0.8%)	0 (0.0%)	0 (0.0%)
Omeprazole	17 (0.2%)	4 (0.5%)	9 (5.4%)

The percentages are calculated from the number of patients in each CKD group (group A; n = 8213, group B; n = 730, group C; n = 166)

### The extent of PPI prescription

**Fig 1:**
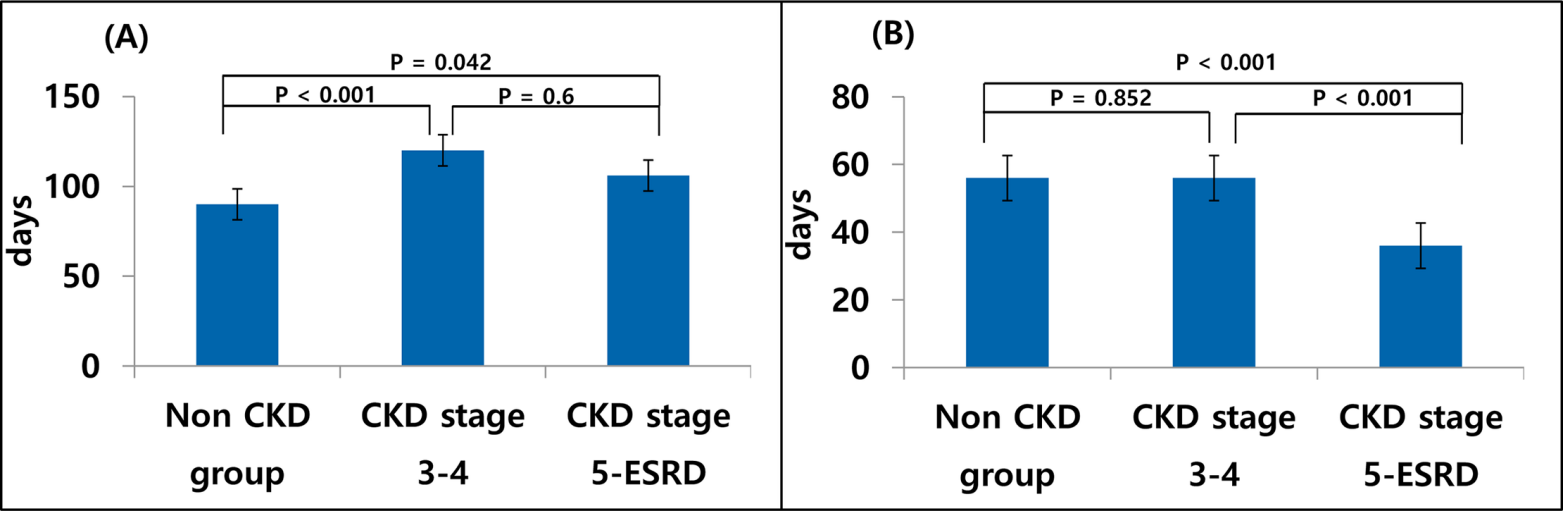
**Median values of total duration of PPI prescription during 2014–2015 by CKD groups (A), and duration prescribed at once by CKD groups(B).** Fig 1A shows the CKD groups were prescribed significantly longer duration of PPIs than the non CKD group (p<0.001). Fig 1B shows the CKD stage5-ESRD group had significantly shorter duration of PPI prescribed at once than the other groups (p<0.001). †pair-wised comparisons were performed and p-value adjusted by bonfferoni correction.

The median duration [95% confidence interval (CI)] of total PPI prescription was 120 [63–273] days in the CKD stage 3–4 group, 106 [56–272] days in CKD stage 5-ESRD group and 90 [56–175] days in non CKD group (Fig 1A). Median duration of PPI prescription for patients with appropriate indication was 67 [56–119] days and for patients without was 91 [56–210] days. When comparing the mean durations, CKD stage 3–4 patients were prescribed PPIs for 1.5 times longer than the non CKD group. Median and mean values showed the CKD group took longer duration of PPIs than the non-CKD group even after adjusting for age and sex (p<0.001). Similarly, median duration of PPI taken consecutively was longest in the CKD stage 3–4 group and shortest in non CKD group (Fig 1B) after adjusting for age and sex (p<0.001). So the CKD stage 3–4 patients not only were prescribed the longest duration of PPIs during the period, but also were the longest duration at once. The patients without known eGFR were excluded in this analysis.

Next, we counted the number of medicine prescribed together with PPIs. Compared to the non-CKD group, the CKD stage 3–4 and CKD stage 5-ESRD groups were prescribed a larger number of drugs simultaneously (6.90±4.17 vs 4.54±2.43; p<0.001, 5.64±2.87 vs 4.54±2.34; p<0.001, respectively).

### Simultaneously prescribed medications

We analyzed the types of medicine prescribed with the PPIs and whether they included medicine such as NSAIDs, steroids, anticoagulants or antiplatelet agents that are often prescribed with PPIs. Among all patients prescribed PPIs, 832 (9.1%) were prescribed NSAIDs, 353 (3.9%) were prescribed steroids and 1,705 (18.7%) were prescribed anticoagulants or antiplatelet agents simultaneously with PPIs. As renal function decreased, likelihood of being prescribed NSAIDs tended to decrease, showing significance in linear by linear association (p<0.001). Patients who were prescribed anticoagulants or antiplatelet agents with PPIs were more likely to be in the CKD groups (Table 3).

**Table 3:** Medicine prescribed with PPIs.

	Non CKD group	CKD 3–4 group	CKD 5-ESRD group
NSAIDs	769 (9.4%)	52 (7.1%)	11 (6.6%)
Steroids	295 (3.6%)	52 (7.1%)	6 (3.6%)
Anticoagulant/antiplatelet	1362 (16.6%)	277 (37.9%)	66 (39.8%)

PPIs, proton-pump inhibitors; NSAIDs, non-steroidal anti-inflammatory drugs

The percentages are calculated from the total number of patients who received PPIs (N = 9109)

### Main departments prescribing PPIs

**Fig 2:**
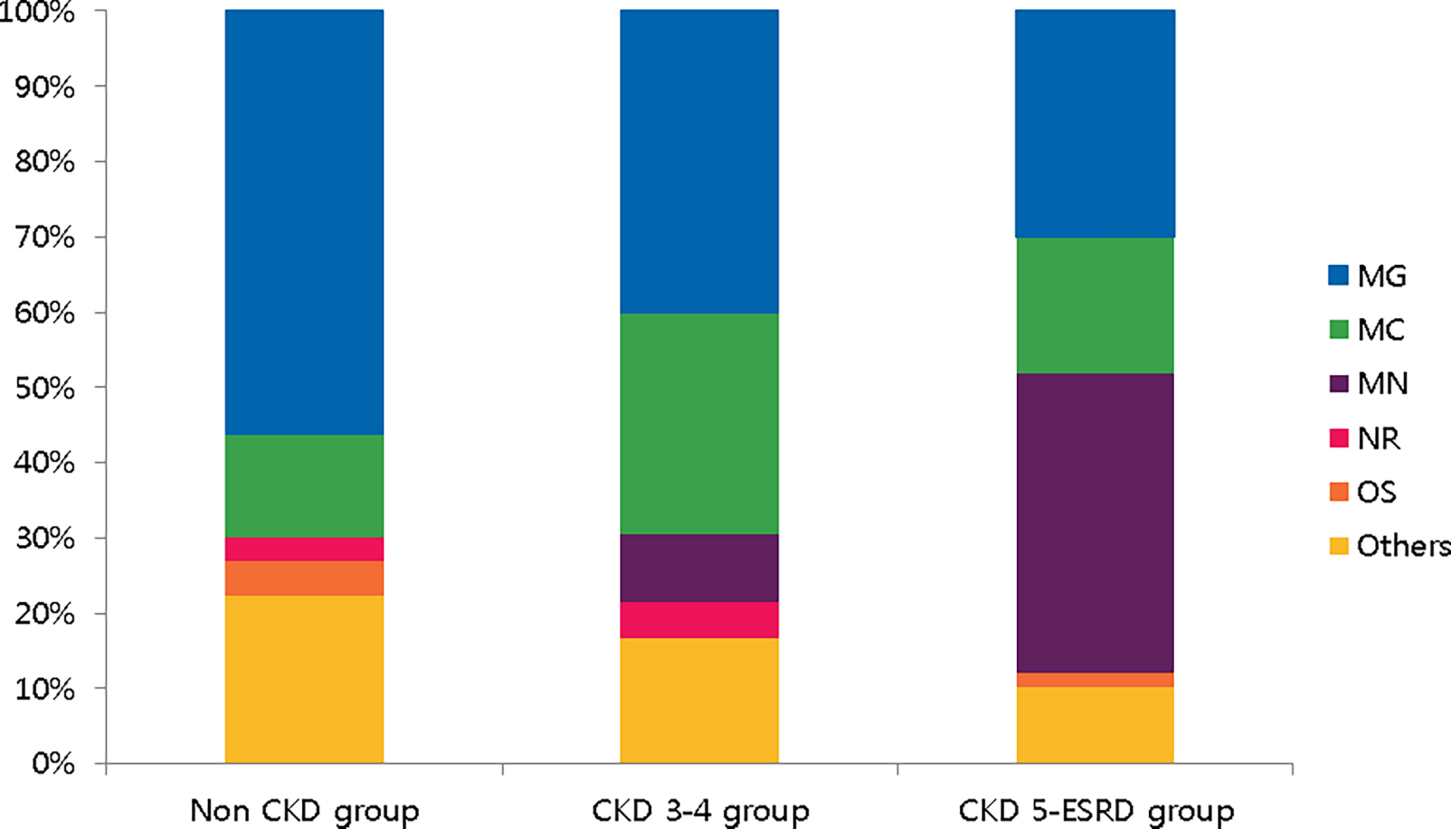
Main departments prescribing PPIs. The percent of prescriptions from the gastroenterology department gradually decreased, while percent of prescriptions from the nephrology department gradually increased as the eGFR decreased (Both p<0.001 in linear by linear association).OS, orthopedic surgery department; MN, Nephrology department; MC, Cardiology department; MG, Gastroenterology department; NR, Neurology department.

The main departments which prescribed PPIs for the CKD stage 3–4 group were gastroenterology (40.0%) and cardiology (29.6%). In the CKD stage 5-ESRD group, nephrologists and gastroenterologists were the main prescribers of PPIs. The gastroenterology, cardiology, and orthopedic surgery departments mainly prescribed PPIs to the non-CKD group (Fig 2). The percentage of PPI prescriptions coming from the nephrology department increased moving from the non-CKD to the CKD stage 3–4 to the CKD stage 5- ESRD groups.

## Discussion

This study showed the patients with CKD stage 3–4 were prescribed more PPIs. Also, more medications were prescribed with PPIs in the CKD groups compared to the non-CKD group. Cardiologists and nephrologists were the main prescribers of PPIs in CKD patients. Nevertheless, it is uncertain that indications for and duration of PPIs prescription have clinical relevance.

A previous study found that the hazard ratio for decline of eGFR over 30% in patients prescribed PPIs for 91–180 days was 2.31 and hazard ratio for developing ESRD or an over 50% decline in eGFR was 1.57 compared to patients prescribed PPIs for less than 30 days over the studies 2 year duration [10]. In our observational study with the same duration, median value of total duration of PPI prescription in the CKD stage 3–4 group was 120 days, which might be long enough to increase risk of CKD progression or risk of ESRD.

There are several reasons why CKD patients take more PPIs than non-CKD patients. First, CKD is associated with increased incidence of acid-related gastrointestinal disorders [18]. Several previous studies showed uremic patients have high prevalence of reflux disease and peptic ulcer disease due to reduction in mucosal prostaglandin, hyperacidity resulting from hypergastrinemia [19], and high incidence of psychological stress and H. pylori infection [20]. Also, there was high recurrence of H. pylori infection after eradication in ESRD patients [21]. Therefore, CKD and ESRD patients may have clinical situations which need PPI treatment.

Secondly, PPIs are frequently prescribed together with medicines such as NSAIDs or anticoagulants and antiplatelet agents which are frequently used in CKD patients. They are prescribed together in order to prevent gastrointestinal (GI) bleeding or ulcerative diseases [22]. Some studies show use of oral anticoagulation is associated with increased risk of GI bleeding, and many physicians tent to prescribe with PPIs [23]. Due to their higher risk of cardiovascular [24] and cerebral vascular diseases [25], a larger proportion of patients with CKD are taking anticoagulants or antiplatelet agents and therefore taking larger amounts of PPIs for longer than patients without CKD. This is also showed in our results. Martin-Echevarria et al. argued gastrointestinal bleeding prophylaxis in administration of anticoagulation or antiplatelet agent was the most frequent inappropriate reason for using PPIs therapy [2]. They suggested the indication for PPIs in this situation is unclear. Also, chronic pain is reported to be prevalent in ESRD and non-dialysis CKD patients and NSAIDs prescription and use is common. However, our results showed NSAIDs were prescribed with PPIs less often in the CKD groups. This may have been due to widespread perceptions about the hazard of NSAIDs in renal impairment [26]. However, it is also possible that other doctors could have been prescribing the NSAIDs or CKD patients were taking over-the-counter NSAIDs.

After analyzing the departments prescribing PPIs, the gastroenterology department was the most dominant in the non-CKD and CKD stage 3–4 groups. However, the cardiology department accounted for dramatically larger proportion of prescriptions in the CKD stage 3–4 group than the non CKD group. Also, the neurology department accounted for 4.8% of CKD stage 3–4 group prescriptions. Some patients in these groups may have received PPIs with antiplatelet/anticoagulant agents for prevention of GI bleeding, which could contribute to overutilization of PPIs.

According to the ICD-10 codes from the electronic medical records of our cohort, a small number of patients a diagnostic code of an appropriate indication for PPIs, and a considerable number of patients were prescribed PPIs with NSAIDs or anticoagulants. Even though some cases, including primary prevention for NSAID-induced ulcerative disease, may be appropriate, prescription patterns of physicians in our study showed PPIs are excessively prescribed to CKD patients and a considerable number of prescriptions lacked appropriate indications. Because studies showing the possible nephrotoxicity of PPIs have only been published recently, the over utilization of PPIs is not surprising in the CKD and non-CKD groups. Regarding the increased awareness of the nephrotoxicity of these drugs, there should be decreased the utilization of PPIs in CKD patients and those at high risk for CKD, and PPIs prescription should be restricted to appropriate indications.

There are several limitations of this study. First, we classified CKD group using creatinine based eGFR. This eGFR was only measured on a single day, so it is possible that some patients with acute kidney injury (AKI) were categorized as having CKD. Also, proteinuria was not included as part of the CKD diagnosis. However, patients with notable changes in renal function would likely to undergo examination or would be hospitalized, so the percentage of subjects misclassified should be negligible. Also, we used ICD-10 codes from the patients’ medical records to reinforce the CKD classification. Second, the duration of the PPI prescription was measured, but the dose of the individual PPIs was not analyzed. This was because prescribing patterns differ among doctors even at the same doses, and there was risk of misreporting of the exact dosage. Third, the number and types of medicines prescribed with PPIs were only counted when prescribed by same department on a same day. Medicines prescribed on another day or prescribed by a different department were not identified. Fourth, exposure to PPIs was calculated from prescriptions in the outpatient setting. Some patients may have skipped their medications. Therefore, actual exposure could be slightly different from what was prescribed for both the CKD and non-CKD groups. Last, there were quite a few people who had no eGFR data. This was inevitable because not all patients require laboratory tests, and our study was based on medical records. But we tried to reduce error by excluded the patients without eGFR data in age, sex weighted comparing. In conclusion, physicians prescribe much longer durations of PPIs to CKD stage 3–4 patients despite the potential nephrotoxic effect of the drugs. Every medication should be cautiously considered for side or unexpected effects to reduce progression of underlying diseases such as CKD. Physicians should pay attention to and consider appropriate indications when prescribing PPIs to CKD patients, especially patients at high risk for aggravation of renal impairment.

## Supporting information

S1 Table Data of the patients enrolled in the study. Age, sex, chronic illnesses (including hypertension, diabetes mellitus, cardiovascular disease, chronic obstructive pulmonary disease, congestive heart failure, liver cirrhosis), CKD groups and laboratory results are included.
(XLSX)
