# Correction: Chronic kidney disease (CKD) patients are exposed to more proton pump inhibitor (PPI)s compared to non-CKD patients

**DOI:** 10.1371/journal.pone.0207561

**Published:** 2018-11-12

**Authors:** Hee Jeong Lee, Haekyung Lee, Song Hee Oh, Joonbyung Park, Suyeon Park, Jin Seok Jeon, HyunJin Noh, Dong Cheol Han, Soon Hyo Kwon

There are errors in Table 1. There are multiple incorrect abbreviations in the first column. Please see the corrected Table 1 here.

**Table 1:** Baseline characteristics of CKD stage 3–4, CKD stage 5-ESRD, and non-CKD groups.

Characteristi*c*s	Non-CKD group (A)	CKD stage 3–4 group (B)	CKD stage 5-ESRD (C)	P value	Post-hoc†
Total	8213 (90.2%)	730 (8.0%)	166 (1.8%)		
Mean age (years)	58.08±14.15	72.30±12.10	62.45±13.20	0.0001	A<C<B
Gender (Male %)	4115 (50.1%)	331 (45.3%)	80 (48.1%)	0.044	A>B>C
Presence of chronic illness (% by groups)					
HTN	1864 (22.7%)	413 (56.6%)	95 (57.2%)	<0.0001	A<B<C
DM	1462 (17.8%)	300 (41.1%)	83 (50.0%)	<0.0001	A<B<C
CVD	987 (12.0%)	216 (29.6%)	51 (30.7%)	<0.0001	A<B<C
LC	179 (2.2%)	28 (3.8%)	9 (5.4%)	0.001	A<B<C
CHF	109 (1.3%)	70 (9.6%)	17(10.2%)	0.0001	A<B<C
COPD	78 (0.9%)	17 (2.3%)	2 (1.2%)	0.002	A<C<B
eGFR (ml/min/1.73 m^2^)	92.78±14.86	44.49±12.06	7.33±3.19	<0.0001	C<B<A

CKD, chronic kidney disease; ESRD, end-stage renal disease; eGFR, estimated glomerular filtration rate; HTN, hypertension; DM, diabetes mellitus; CVD, cardiovascular disease; LC, liver cirrhosis; CHF, congestive heart failure; COPD, chronic obstructive pulmonary disease. eGFR was calculated using CKD-EPI formula. Age and eGFR are presented as mean ± standard deviation (SD). Nominal data are presented as percentages

†p-value by student t-test or chi-squared/fisher exact test and adjusted by bonferroni correction
